# Immune system benefits of pulmonary rehabilitation in chronic obstructive pulmonary disease

**DOI:** 10.1113/EP091678

**Published:** 2024-10-25

**Authors:** Asghar Abbasi, David Wang, William W. Stringer, Richard Casaburi, Harry B. Rossiter

**Affiliations:** ^1^ Institute of Respiratory Medicine and Exercise Physiology The Lundquist Institute for Biomedical Innovation at Harbor‐UCLA Medical Center Torrance California USA

**Keywords:** COPD, exercise training, immune system, inflammation, pulmonary rehabilitation

## Abstract

Chronic obstructive pulmonary disease (COPD) is a respiratory disease characterized by pulmonary and systemic inflammation. Inflammatory mediators show relationships with shortness of breath, exercise intolerance and health related quality of life. Pulmonary rehabilitation (PR), a comprehensive education and exercise training programme, is the most effective therapy for COPD and is associated with reduced exacerbation and hospitalization rates and increased survival. Exercise training, the primary physiological intervention within PR, is known to exert a beneficial anti‐inflammatory effect in health and chronic diseases. The question of this review article is whether exercise training can also make such a beneficial anti‐inflammatory effect in COPD. Experimental studies using smoke exposure mice models suggest that the response of the immune system to exercise training is favourably anti‐inflammatory. However, the evidence about the response of most known inflammatory mediators (C‐reactive protein, tumour necrosis factor α, interleukin 6, interleukin 10) to exercise training in COPD patients is inconsistent, making it difficult to conclude whether regular exercise training has an anti‐inflammatory effect in COPD. It is also unclear whether COPD patients with more persistent inflammation are a subgroup that would benefit more from hypothesized immunomodulatory effects of exercise training (i.e., personalized treatment). Nevertheless, it seems that PR combined with maintenance exercise training (i.e., lifestyle change) might be more beneficial in controlling inflammation and slowing disease progress in COPD patients, specifically in those with early stages of disease.

## Abstract (highlights)

- **What is the topic of this review?**
  The response of immune cells and inflammatory markers to exercise training in cigarette smoke exposure and COPD.
- **What advances does it highlight?**
  Exercise training modifies pulmonary inflammation and slows development or progression of emphysema in smoke‐exposed mice, but evidence is lacking to support immunomodulation by pulmonary rehabilitation in COPD patients.

## 1 INTRODUCTION

Chronic obstructive pulmonary disease (COPD) is a major public health problem primarily associated with long‐term exposure to toxic gases or particulates, which, in Western society, is mostly caused by tobacco smoke exposure. Primary host defence mechanisms against this insult include innate and adaptive inflammatory responses in the lungs. Disease processes in COPD are characterized by complex interplay of several factors, including oxidative stress, inflammation, tissue damage and impaired tissue repair mechanisms. Macrophages, neutrophils, and T lymphocytes each contribute to inflammation, whereby chronic cigarette smoke (CS) exposure leads to chronic inflammation. COPD pathology is characterized by emphysema and small‐airway remodelling, excessive mucus production and progressive loss of lung function. Most patients present with a mixed picture of small airways disease and emphysema, although some patients may show a predominance of one or the other.

COPD exacerbations are the most significant driver of health decline and healthcare use in COPD. COPD exacerbations are typically triggered by respiratory viral or bacterial infections (Fabbri et al., 2004), and a distinct group of patients appear more susceptible to frequent exacerbations, irrespective of the degree of lung dysfunction (Carney et al., 2020). Chronic inflammation of the respiratory tract, mostly derived from activated macrophages and neutrophils, is further increased during periods of COPD exacerbation, which accompanies heightened symptoms of dyspnoea and may require treatment (antibiotics, corticosteroids) or hospitalization and, in extremis, can lead to death (Sethi et al., 2000).

Although COPD treatments have advanced, there is still a significant unmet need to identify approaches that slow the disease course, reduce exacerbations of COPD and lower mortality rates. While a few available drugs (e.g., inhaled corticosteroids, monoclonal antibodies) may slow COPD progression and decrease exacerbations (Bhatt et al., 2023; Lipson et al., 2018; Loncharich & Anderson, 2022; Rabe et al., 2020), no medications are without side effects. Pulmonary rehabilitation (PR), a comprehensive education and exercise training programme, is one of the most effective therapies for improving symptoms and outcomes in COPD. Completion of PR within 1–6 months of hospitalization for acute exacerbation of COPD (AECOPD) reduces hospital readmissions (Puhan et al., 2016) and mortality risk (Lindenauer et al., 2020). In health, exercise training exerts a beneficial systemic immunomodulatory effect (Gleeson et al., 2011; Lancaster & Febbraio, 2014). In this review, we investigate the impact of CS exposure and/or COPD on the immune system, and whether immunomodulation by exercise training (the primary physiologic intervention within PR) may mediate a reduced risk for AECOPD or mortality in COPD patients.

## 2 THE ANTI‐INFLAMMATORY EFFECTS OF EXERCISE TRAINING

In health, a large body of evidence demonstrates that lack of physical activity or physical fitness is associated with increased risk of cardiovascular disease, stroke, cancer, diabetes and other chronic diseases (Booth et al., 2012; Gleeson et al., 2011). Inflammation is aetiologically linked to the pathogenesis of each of these conditions (Hotamisligil, 2006; Ouchi et al., 2011; Rook & Dalgleish, 2011; Shoelson et al., 2006), and chronic low‐grade systemic inflammation is associated with disease severity in many (Pradhan et al., 2001). Regular physical activity reduces risk of chronic metabolic and cardiorespiratory diseases, which may, at least in part, be ascribed to an anti‐inflammatory effect of regular physical activity. These effects are proposed to be mediated by reduction in visceral fat mass and associated decreased inflammatory adipokine release, as well as induction of an anti‐inflammatory environment associated with release of exercise‐induced ‘exerkines’ and/or muscle‐derived ‘myokines’, such as interleukin (IL)‐6 (Chow et al., 2022; Pedersen, 2009). Circulating IL‐6 is acutely increased in exercise (Fischer, 2006; Meckel et al., 2009), which promotes an increase in circulating IL‐10, interleukin 1 receptor antagonist (IL‐1ra) and cortisol (Steensberg et al., 2003). IL‐10 suppresses monocyte production of tumour necrosis factor α (TNF‐α; Opp et al., 1995), and IL‐1ra inhibits IL‐1β signal transduction (Bergmann et al., 1999; Dinarello, 1994; Gleeson et al., 2006). In this way, regular physical activity or exercise training promotes chronic adaption of immune system function towards a more anti‐inflammatory phenotype (Aw et al., 2018; Gleeson et al., 2011; Nieman & Wentz, 2019; Walsh et al., 2011).

Amelioration of toll like receptor (TLR)‐dependent inflammation is also considered a key mechanism by which exercise training modifies the immune system (Gleeson et al., 2006; Oliveira & Gleeson, 2010). TLRs are a family of transmembrane receptors central to innate immunity (Kawai & Akira, 2010). TLR2 and TLR4 play a key role in development of many chronic diseases, including COPD (Barnes, 2017). In health and across ageing, exercise training decreases TLR4 expression on the surface of circulating monocytes as well as expression of nuclear factor κB (NF‐κB) mRNA (Flynn et al., 2003; Lancaster et al., 2005; Liu & Chang, 2018; McFarlin et al., 2004). High intensity exercise also increases negative regulators of NF‐κB in athletes (Abbasi et al., 2014). NF‐κB, a redox‐sensitive transcription factor downstream of TLRs, is involved in a wide variety of inflammatory networks that control cytokine activity in tissue pathology, including airway pathology (Schuliga, 2015). Furthermore, exercise training also decreases number and infiltration of immune cells into tissues and promotes phenotypic switching of macrophages from inflammatory (M1) towards the anti‐inflammatory (M2) phenotype (Kawanishi et al., 2010; Kawanishi et al., 2015; Ruffino et al., 2016; Suzuki et al., 1996; Takahashi et al., 2013; Yakeu et al., 2010). In athletes who participate in exercise training (in particular high intensity exercise training), increased circulating numbers of regulatory T cells (Tregs), well‐known anti‐inflammatory T cells, also contribute to the anti‐inflammatory state (Weinhold et al., 2016). Finally, reduced neutrophil reactive oxygen species (ROS) production and increased antioxidant defences are also considered as anti‐inflammatory mechanisms of exercise training (Fatouros et al., 2004; Vezzoli et al., 2014). Taken together, regular long‐term exercise can protect against development of chronic diseases through its anti‐inflammatory effects.

## 3 IMMUNE SYSTEM DERANGEMENTS AND INFLAMMATION IN COPD

**FIGURE 1:**
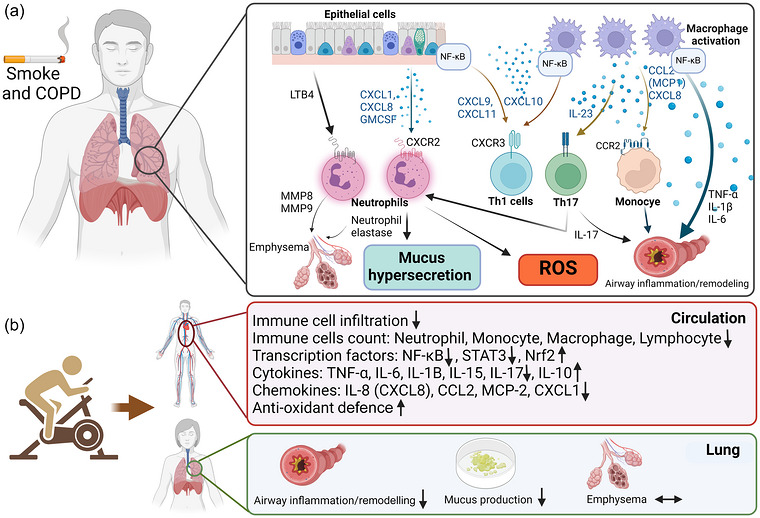
Induction of inflammation by cigarette smoke inhalation and COPD (a), and suppression of systemic and lung tissue inflammation by exercise training in smoke‐exposed COPD (b).

Tobacco smoke, or other toxic irritants, causes airway epithelial damage and phenotypic changes in lung epithelial cells, such as squamous metaplasia (Aghapour et al., 2018). Injured lung epithelial cells release cytokines (including alarmins), chemokines, adhesion molecules and growth factors to repair epithelium and/or recruit inflammatory cells, which also modulate other airway wall components and immune cell gene expression (Kearley et al., 2015; Barnes, 2017). Resulting chemotactic factors released from epithelial cells (e.g., chemokine ligand (CXCL1), CXCL9, CXCL8/IL‐8, CXCL10, CXCL11 and granulocyte–macrophage colony‐stimulating factor (GM‐CSF)) and macrophages (e.g., CCL2/monocyte chemoattractant protein 1 (MCP‐1), IL‐23, CXCL10, and CXCL8/IL‐8) recruit neutrophils and monocytes (which later differentiate into macrophages) to the lung (Barnes, 2017; Barnes, 2019) (Figure 1a). Neutrophils and macrophages aggregate to airway lumen, and macrophages, T lymphocytes and B lymphocytes to airway wall and parenchyma. Thus, the inflammatory response of both innate and adaptive immune systems to CS exposure contributes to the pathogenesis of COPD (Barnes, 2017). Recruitment of type 1 cytotoxic T cells (Tc1) and T helper cells (Th1) (Saetta et al., 1999), together with activated innate lymphoid cells (ILC1 and ILC3), likely contribute to sustaining neutrophilic inflammation in COPD (Costa et al., 2008; Barnes, 2019). Activated immune cells, particularly macrophages, and epithelial cells release proteases such as neutrophil elastase and matrix metalloproteinase 9 (MMP‐9), which cause elastin breakdown and contribute to the development of pulmonary emphysema. Increased neutrophil elastase in lungs of people with COPD also results in mucus hypersecretion. Epithelial cells and macrophages release transforming growth factor β (TGF‐β), which stimulates fibroblast proliferation, promoting fibrosis in small airways (Barnes, 2008). Each of these events contributes to chronic inflammation and pulmonary remodelling in COPD, with downstream consequences of loss of lung elastic recoil, expiratory flow limitation, and shortness of breath on exertion.

COPD is also characterized by chronic systemic inflammation, which may be supplemented by spillover from chronic pulmonary inflammation. Systemic inflammation itself is associated with greater lung function decline and worse clinical outcomes (Barnes, 2017). Systemic inflammation in COPD is characterized by elevated concentration of circulating cytokines (e.g., IL‐4, IL‐6, TNF‐α, leptin), chemokines (e.g., CXCL8/IL‐8, CCL4, CCL22, CCL2) and acute phase proteins (e.g., C‐reactive protein (CRP) and serum amyloid A (SAA)), together with neutrophilia (Agustí et al., 2012; Bruzzaniti et al., 2019; Foschino Barbaro et al., 2007; Gan et al., 2004; Kim et al., 2015). In a large population study, involving 2164 COPD patients, white blood cells (WBCs), serum CRP, IL‐6 and fibrinogen were each greater in COPD patients than in smokers with normal lung function or non‐smokers (Agustí et al., 2012). These factors (but not CXCL8/IL‐8 or TNF‐α) tended to increase with severity of expiratory flow limitation (Agustí et al., 2012). Moreover, during a 3‐year follow‐up, both adjusted all‐cause mortality and adjusted annual rate of AECOPD were greater in individuals with persistent systemic inflammation compared to those without, independent of rate of lung function decline (Agustí et al., 2012).

Blocking of inflammatory mediators remains an active target for new COPD therapies. However, prior attempts to produce therapeutic antagonists for several cytokines and chemokines provided relatively little clinical benefit, despite reducing sputum inflammatory cell numbers (Rennard & Drummond, 2015). The reason for failure of these potential therapies is thought to be the multiplicity and redundancy inherent in the immune system, implying that preventing synthesis or action of a single mediator is unlikely to yield major clinical benefit on COPD outcomes. In addition, high incidence of comorbidities in COPD patients, such as cardiovascular disease, diabetes and obesity, are also associated with chronic low‐grade inflammation and physical inactivity, which may contribute to the complexity in targeting COPD‐associated inflammation as a potential treatment. A recent study showed that a subset of COPD patients with type 2 inflammation who received dupilumab, a fully human monoclonal antibody IL‐4 receptor α‐agonist that results in the blockade of both IL‐4 and IL‐13 signalling, had fewer AECOPD, improved lung function and quality of life, and milder respiratory symptoms than those who received placebo (Bhatt et al., 2023). Therefore, development of effective anti‐inflammatory therapies for COPD treatment should act on a broad spectrum of inflammatory mediators. Effective anti‐inflammatory therapies are probably the greatest unmet need in COPD.

## 4 EFFICACY OF PR IN COPD

PR is likely the most efficacious therapy in COPD for reducing symptoms, AECOPD and mortality, but less than ∼5% of eligible patients currently receive PR (Desveaux et al., 2015; Nishi et al., 2016). Physical activity is dramatically reduced in COPD patients, especially in moderate or severe COPD, and further decline in physical activity is paralleled by worsened lung function and health status (Waschki et al., 2015). PR reduces disease symptoms and AECOPD while improving quality of life (Garcia‐Aymerich et al., 2007; Laveneziana & Palange, 2012). PR also reduces the risk of hospital readmissions compared with usual care (odds ratio (OR) = 0.44; 95% CI: 0.12, 0.91) (Puhan et al., 2016), and PR initiation is associated with increased survival (every three additional PR sessions completed reduces risk of death; hazard ratio (HR) = 0.91; 95% CI: 0.85, 0.98) (Lindenauer et al., 2020). Thus, increasing physical fitness through PR represents a major opportunity to reduce chronic inflammation, slow disease progression, reduce exacerbations and increase quality of life in COPD patients.

## 5 EFFECTS OF PR ON IMMUNE SYSTEM FUNCTION IN COPD

It is well established that 6–12 weeks of exercise training in COPD increases strength and exercise tolerance. However, clinical studies repeatedly demonstrate that chronic low‐grade systemic inflammation is also associated with physical inactivity in COPD, even after adjusting for relevant covariates (Garcia‐Aymerich et al., 2009; Moy et al., 2014; Waschki et al., 2012; Watz et al., 2008). It is not surprising, therefore, that evidence supports a reduction of the number of circulating inflammatory cells and mediators in COPD patients following PR (Jenkins et al., 2020) (Table 1, Figure 1b). Here we review pre‐clinical and clinical evidence that exercise training, for example, as part of a PR programme, modifies the immune system, immune function and inflammation in cigarette smoke‐induced COPD.

**TABLE 1:** Inflammatory/immune system biomarkers in smoke exposure or COPD and their response to exercise training.

		Effect of smoke exposure or COPD			Effect of exercise training in smoke exposure or COPD		
Biomarker	Tissue	Direction	Species	References	Direction	Species	References
WBCs/total cells	Blood, BALF, lung	↑	Mouse, human	Kubo et al., 2019; Rodrigues Brandao‐Rangel et al., 2017; Ryrsø et al., 2018; Toledo‐Arruda et al., 2017; Van Helvoort et al., 2006; Yu et al., 2012	↔↓	Mouse, human	Jenkins et al., 2020; Neunhäuserer et al., 2021; Rodrigues Brandao‐Rangel et al., 2017; Sciriha et al., 2017; Yu et al., 2012
Neutrophils	Blood, BALF	↑	Mouse, human	Cervilha et al., 2019; Kubo et al., 2019; Menegali et al., 2009; Rodrigues Brandao‐Rangel et al., 2017; Ryrsø et al., 2018; Toledo‐Arruda et al., 2017; Yu et al., 2012	↔↓	Mouse, human	Jenkins et al., 2020; Kubo et al., 2019; Menegali et al., 2009; Rodrigues Brandao‐Rangel et al., 2017; Sciriha et al., 2017; Yu et al., 2012
Monocytes	Blood	↑	Mouse	Rodrigues Brandao‐Rangel et al., 2017	↓	Mouse	Rodrigues Brandao‐Rangel et al., 2017
Macrophages	BALF, lung	↑	Mouse	Menegali et al., 2009; Toledo et al., 2012; Yu et al., 2012; Cervilha et al., 2019	↔↓	Mouse	Menegali et al., 2009; Toledo et al., 2012; Yu et al., 2012
Lymphocytes	Blood, BALF	↑	Mouse	Rodrigues Brandao‐Rangel et al., 2017; Toledo‐Arruda et al., 2017; Yu et al., 2012	↓	Mouse	Yu et al., 2012; Rodrigues Brandao‐Rangel et al., 2017
Eosinophils	Blood, lung, and BALF	↓	Mouse	Botelho et al., 2011; Melgert et al., 2004; Robbins et al., 2005	↔↓	Human	Neunhäuserer et al., 2021; Sciriha et al., 2017
CRP	Blood	↑	Human	Celli et al., 2019; Petersen et al., 2008; Ryrsø et al., 2018; Van Helvoort et al., 2006; Vogiatzis et al., 2007	↔↓	Human	Gelinas et al., 2017; Jenkins et al., 2020; Kantorowski et al., 2018; Márquez‐Martín et al., 2014; Neunhäuserer et al., 2021; Petersen et al., 2008; Ryrsø et al., 2018; Sciriha et al., 2017; Vogiatzis et al., 2007; Wang et al., 2014
Fibrinogen	Blood	↑	Human	Celli et al., 2019	↓	Human	Jenkins et al., 2020; Neunhäuserer et al., 2021
IL‐15	Blood	↓	Human	Mian et al., 2009	↑	Human	Silva et al., 2018
IL‐18	Blood	↑	Human	Petersen et al., 2008; Ryrsø et al., 2018	↔	Human	Petersen et al., 2008; Ryrsø et al., 2018
ICAM‐1	Lung, lymphocytes	↑	Mouse	Krüger et al., 2018; Yu et al., 2012	↔↓	Mouse Human	Gelinas et al., 2017; Krüger et al., 2018; Yu et al., 2012
VCAM‐1	Lung, lymphocytes	↑	Mouse	Krüger et al., 2018; Yu et al., 2012	↔↓	Mouse Human	Krüger et al., 2018; Yu et al., 2012
Nrf2	Lung, muscle	↔↓	Mouse	Nemmar et al., 2018; Kubo et al., 2019	↑	Mouse	Kubo et al., 2019; Nemmar et al., 2018; Toledo‐Arruda et al., 2020
NF‐κB	Lung	↑	Mouse	Nemmar et al., 2018; Yu et al., 2012	↓	Mouse	Nemmar et al., 2018; Yu et al., 2012
STAT3	Airway epithelium, leukocytes	↑	Mouse	Cervilha et al., 2019; Rodrigues Brandao‐Rangel et al., 2017	↓	Mouse	Rodrigues Brandao‐Rangel et al., 2017
TNF‐α	Blood, BALF, lung, muscle	↑	Mouse, human	Bolton et al., 2007; Krüger et al., 2018; Nemmar et al., 2018; Peñailillo et al., 2023; Rabinov et al., 2003; Rodrigues Brandao‐Rangel et al., 2017; Toledo‐Arruda et al., 2017; Toledo‐Arruda et al., 2020; Vogiatzis et al., 2007	↔↓	Mouse, human	Bolton et al., 2007; Gelinas et al., 2017; Krüger et al., 2018; Nemmar et al., 2018; Neunhäuserer et al., 2021; Petersen et al., 2008; Rabinov, 2003; Rodrigues Brandao‐Rangel et al., 2017; Silva et al., 2018; Toledo‐Arruda et al., 2017; Vogiatzis et al., 2007; Toledo‐Arruda et al., 2020; Wang et al., 2014
IL‐1β	Blood, BALF, muscle	↑	Mouse, human	Yu et al., 2012; Rodrigues Brandao‐Rangel et al., 2017; Krüger et al., 2018	↓	Mouse	Yu et al., 2012; Rodrigues Brandao‐Rangel et al., 2017; Krüger et al., 2018
IL‐6	Blood, BALF	↑↔	Mouse, human	Bolton et al., 2007; Cervilha et al., 2019; Muller, 2003; Nemmar et al., 2018; Rodrigues Brandao‐Rangel et al., 2017; Van Helvoort et al., 2006; Vogiatzis et al., 2007	↑↔↓	Mouse, human	Bolton et al., 2007; da Silva et al., 2017; do Nascimento et al., 2015; Gelinas et al., 2017; Nemmar et al., 2018; Neunhäuserer et al., 2021; Petersen et al., 2008; Rodrigues Brandao‐Rangel et al., 2017; Silva et al., 2018; Toledo‐Arruda et al., 2017; Vogiatzis et al., 2007; Wang et al., 2014
IL‐8 (CXCL8)	Blood, BALF, sputum	↑	Mouse, human, T cells	do Nascimento et al., 2015; Drost et al., 2005; Oltmanns et al., 2005; Ryrsø et al., 2018; Van Helvoort et al., 2006)	↔↓	Human	do Nascimento et al., 2015; Gelinas et al., 2017; Márquez‐Martín et al., 2014; Ryrsø et al., 2018; Szczegielniak et al., 2011; Uzeloto et al., 2022; Wang et al., 2014)
IL‐10	Blood, BALF, lung, muscle	↔↓	Mouse	Cervilha et al., 2019; Rodrigues Brandao‐Rangel et al., 2017; Toledo et al., 2012; Toledo‐Arruda et al., 2020)	↑↔	Mouse, human	Rodrigues Brandao‐Rangel et al., 2017; Silva et al., 2018; Toledo et al., 2012; Toledo‐Arruda et al., 2017; Toledo‐Arruda et al., 2020; Uzeloto et al., 2022)
IL‐17	Blood, BALF	↑↔	Mouse	Cervilha et al., 2019; Rodrigues Brandao‐Rangel et al., 2017)	↓	Mouse	Rodrigues Brandao‐Rangel et al., 2017)
MCP‐1 (CCL2)	BALF, parenchyma	↑	Mouse	Cervilha et al., 2019; Toledo et al., 2012; Yu et al., 2012	↓	Mouse	Toledo et al., 2012; Yu et al., 2012
MCP‐2	Lung	↑	Mouse	Yu et al., 2012	↓	Mouse	Yu et al., 2012
CXCL1	Blood, BALF	↑	Mouse	Cervilha et al., 2019; Rodrigues Brandao‐Rangel et al., 2017	↓	Mouse	Rodrigues Brandao‐Rangel et al., 2017
Mucin 2	Lung	↑	Mouse	Yu et al., 2012	↓	Mouse	Yu et al., 2012
SOD	Lung	↓↑	Mouse	Menegali et al., 2009; Nesi et al., 2016	↑	Mouse	Menegali et al., 2009; Nesi et al., 2016
ROS	BALF	↑↔	Mouse, human	Toledo et al., 2012; Van Helvoort et al., 2006	↓	Mouse, human	Neunhäuserer et al., 2021; Toledo et al., 2012

## 6 EVIDENCE FROM MOUSE STUDIES

There are a wide range of murine COPD models. For specificity, this review will only present data from mouse studies with cigarette smoke‐induced lung disease, due to its wide use in the literature as a model for COPD. However, it is recognized that the mouse inflammatory response to cigarette smoke exposure has limitations as a model system for COPD, and different strains have different responses, suggesting underlying genetic differences. Genetic variation in humans also contributes to the development of smoke‐induced COPD (Lomas & Silverman, 2001). It is widely accepted that cigarette smoke is a potent pro‐inflammatory agent that causes pulmonary inflammation and structural alterations by inducing the over‐production and release of various pro‐inflammatory cytokines and chemokines, as well as increasing immune cell influx to the lungs (Figure 1a, Table 1) (Barnes, 2008; Cervilha et al., 2019). However, evidence from experimental murine studies suggests that administration of an exercise programme during or following smoke exposure reduces systemic and lung inflammation and slows disease progression (Krüger et al., 2018; Menegali et al., 2009; Rodrigues Brandao‐Rangel et al., 2017; Yu et al., 2012). This includes a decrease in circulatory and tissue resident inflammatory cells, as well as reduction in inflammatory cytokines in the circulation, bronchoalveolar lavage fluid (BALF) and lung tissue (IL‐1, IL‐6, IL‐8, IL‐17 and TNF‐α), and modification of epithelial and endothelial function (Bowen et al., 2017; Krüger et al., 2018; Madani et al., 2018; Menegali et al., 2009; Rodrigues Brandao‐Rangel et al., 2017; Toledo‐Arruda et al., 2017; Toledo et al., 2012; Yu et al., 2012) (Table 1).

### 6.1 Effect of exercise training on immune cell infiltration and number

Exercise training decreases CS‐induced leukocyte infiltration in lung tissues (Yu et al., 2012) (Figure 1b). Leukocyte migration to sites of lung inflammation is a characteristic feature of lung inflammation and is facilitated by inflammatory mediators and adhesion molecules (Muller, 2003). In addition, aerobic exercise training decreases WBC number in blood and BALF (Menegali et al., 2009; Rodrigues Brandao‐Rangel et al., 2017; Vieira et al., 2012; Yu et al., 2012). Eight weeks of treadmill running in male C57BL/6 mice (8 m/min for 30 min twice/day, 5 days/week, 8 weeks) prior to CS exposure (4 weeks), significantly reduced pulmonary leukocyte infiltration (Yu, Liao et al., 2012). In male C57BL/6 mice, whereas 12 weeks of CS exposure significantly increased immune cell numbers in BALF, treadmill exercise training attenuated this increase in CS‐exposed mice (Toledo‐Arruda, Vieira et al., 2017). In another study, while CS exposure (90 days) resulted in increased immune cell numbers in blood and BALF, these numbers were significantly lower in mice exposed to both CS and exercise training (treadmill running at 50% of maximal velocity, 25% incline, 60 min/day, 5 days/week, 4 weeks) (Rodrigues Brandao‐Rangel et al., 2017).

### 6.2 Effect of exercise training on inflammatory transcription factors

Several animal studies indicate that aerobic exercise training modifies the inflammatory response by transcription factor modulation (Kubo et al., 2019; Nemmar et al., 2018; Rodrigues Brandao‐Rangel et al., 2017; Yu et al., 2012). Nuclear factor erythroid 2‐related factor (Nrf2) is a transcription factor involved in oxidative stress defence, including damage from CS (Cho & Kleeberger, 2015). Nrf2‐deficient mice are highly vulnerable to CS‐induced lung injury, and Nrf2 expression was significantly decreased in bronchial and alveolar epithelial cells of COPD patients compared to control subjects. This emphasizes the protective role of Nrf2 against smoke‐induced apoptosis and its potential to moderate oxidant–antioxidant imbalance emphysema (Iizuka et al., 2005; Yamada et al., 2016). Kubo et al. evaluated Nrf2 gene expression in lung homogenate of CS‐exposed mice following treadmill running (18 m/min, 0° incline, 30 min/day, 5 days/week, 12 weeks). While no significant difference was observed in Nrf2 mRNA expression between control and CS‐exposed mice, Nrf2 mRNA expression was significantly greater in mice with combined CS exposure and exercise training (Kubo et al., 2019), suggesting a protective adaptation of exercise training reducing CS‐related oxidative damage. Such a protective role of Nrf2 has been previously demonstrated in animal models of oxidant‐related pulmonary disease such as emphysema (Iizuka et al., 2005), acute lung injury (Reddy et al., 2009) and pulmonary fibrosis (Kikuchi et al., 2010) (Figure 1b, Table 1).

Activation of NF‐κB signalling in alveolar macrophages and parenchyma has a pivotal role in promoting inflammation in response to smoke exposure (Caramori et al., 2003; Di Stefano et al., 2002; Pace et al., 2008). The activity of NF‐κB was significantly decreased in lungs of male C57BL/6 mice in response to 8 weeks of treadmill running prior to CS exposure (8 m/min, 30 min twice/day, 5 days/week, 8 weeks) (Yu et al., 2012). In another study, treadmill running (12 m/min, 40 min/day, 5 days/week, 8 weeks) prior to water pipe smoke exposure (2 months) reduced NF‐κB gene expression and elevated Nrf2 protein in lung tissue (Nemmar et al., 2018) (Figure 1b, Table 1).

Signal transducer and activator of transcription 3 (STAT3) is another transcription factor that may mediate anti‐inflammatory benefits of exercise training in the face of CS‐induced lung disease. STAT3 is activated by various mediators, most notably IL‐6, and is associated with pulmonary inflammation and emphysema (McLeod et al., 2012; Ruwanpura et al., 2011). IL‐6, along with other cytokines such as IL‐21 and IL‐23, regulates Th17 cell differentiation by inducing STAT3 and, as a result, increases IL‐17 production (Kimura & Kishimoto, 2010; McGeachy & Cua, 2007). Th17 cells and IL‐17 are involved in development of inflammation and emphysema in CS‐exposed animals (Ruwanpura et al., 2014). Sixty days of CS exposure increased IL‐17 in BALF and serum in mice (Rodrigues Brandao‐Rangel et al., 2017), while treadmill running (50% of maximal velocity, 25° incline, 60 min/day, 5 days/week, 4 weeks) after CS exposure reduced both expression and phosphorylation of STAT3 in airway epithelial cells, peribranchial leukocytes and parenchymal leukocytes (Rodrigues Brandao‐Rangel et al., 2017) (Figure 1b, Table 1).

Overall, these findings provide evidence of potential protective and anti‐inflammatory benefits of exercise training in CS‐exposed mice.

### 6.3 Effect of exercise training on inflammatory mediators

Aerobic exercise training reduces concentration of several cytokines and chemokines (e.g., IL‐1β, TNF‐α, IL‐ 6, IL‐17, CXCL1, MCP‐1, macrophage inflammatory protein 2 (MIP‐2), intercellular adhesion molecule 1 (ICAM‐1), vascular cell adhesion molecule 1 (VCAM‐1) and Mucin 2) in blood and BALF of the smoke‐exposed murine (Table 1) (Rodrigues Brandao‐Rangel et al., 2017; Yu et al., 2012). We showed that smoke‐exposed mice (90 days of CS) had greater concentrations of IL‐1β, TNF‐α, IL‐17 and CXCL1 in BALF and blood (Table 1), whereas combined smoke exposure and exercise training (90 days CS plus 60 days treadmill running at 50% maximal velocity, 25° incline, 60 min/day, 5 days/week, 8 weeks) significantly reduced these mediators (Rodrigues Brandao‐Rangel et al., 2017). Similar effects were observed by Toledo‐Arruda et al. (2017) and Krueger et al. (2018), demonstrating a decrease in lung TNF‐α concentration and reduced expression of surface markers ICAM‐1 and VCAM‐1 on lymphocytes from smoke‐exposed mice, respectively, after exercise training interventions (Krüger et al., 2018; Toledo‐Arruda et al., 2017) (Table 1). Additionally, studies on inflammatory effects of water pipe smoke exposure in mice, simulating human exposure protocols, showed increased concentrations of IL‐6 and TNF‐α in lung homogenates, which were mitigated by treadmill exercise (at 12 m/min, 12% grade, 40 min/day, 5 days/week, 60 days) (Nemmar et al., 2018) (Figure 1b, Table 1). Furthermore, Yu et al. (2012) demonstrated the preventive effect of exercise training against smoke‐induced lung inflammation, showing reductions in IL‐1β, MIP‐1, MIP‐2, ICAM‐1 and VCAM‐1 concentration in lung homogenates following treadmill running (8 m/min, 30 min twice/day, 5 days/week, 8 weeks) (Yu et al., 2012).

Exercise training also attenuates muscle inflammatory response caused by smoke exposure (Krüger et al., 2018; Toledo‐Arruda et al., 2020). Krueger et al. (2018) investigated the effect of treadmill exercise (80% ${\dot{V}}_{O_{2}\max}$, 30 min/day, 5 days/week, 8 weeks) on rectus femoris inflammatory profile using gene expression analysis. They found that exercise training significantly attenuated IL‐1β and TNF‐α gene expression in CS‐exposed mice (Krüger et al., 2018). Exercise training (30 min twice/day, 5 days/ week, up to 12 weeks) also significantly reduced TNF‐α gene expression and increased Nrf2 gene expression in muscle of CS‐exposed mice (Toledo‐Arruda et al., 2020).

In addition to reducing pro‐inflammatory mediators, exercise training may also help mitigate effects of CS on inflammation by increasing production and release of anti‐inflammatory cytokines such as IL‐10 (Rodrigues Brandao‐Rangel et al., 2017; Toledo et al., 2012; Toledo‐Arruda et al., 2017; Toledo‐Arruda et al., 2020). IL‐10 modulates expression of inflammatory cytokines, soluble mediators and myeloid cell surface molecules that influence onset and duration of inflammatory responses (Moore et al., 2001). A few experimental studies have demonstrated that IL‐10 mRNA expression and protein concentration are greater in a range of tissues in exercise‐trained mice compared with CS exposure (Rodrigues Brandao‐Rangel et al., 2017; Toledo et al., 2012; Toledo‐Arruda et al., 2017; Toledo‐Arruda et al., 2020; Vieira et al., 2012). Toledo et al. (2020) found that IL‐10 mRNA expression was significantly greater in skeletal muscle of combined CS exposure and exercise training (30 min twice/day, 5 days/week, for 4, 8, and 12 weeks) compared with CS alone (Toledo‐Arruda et al., 2020). Exercise training also protected against loss of IL‐10‐positive lung tissue cells in CS‐exposed mice (Toledo et al., 2012). Brandao‐Rangel et al. (2017) also reported a lower IL‐10 concentration in both blood and BALF of CS‐exposed mice compared with combined CS and treadmill exercise training (50% maximal velocity, 25° incline, 60 min/day, 5 days/week, 8 weeks) (Rodrigues Brandao‐Rangel et al., 2017). We have previously shown an anti‐inflammatory effect of IL‐10 in cell culture. Human whole blood, endothelial cells and human neutrophils were incubated with IL‐10 before adding lipopolysaccharide (LPS) to the culture. This study also showed that human whole blood, endothelial cells and human neutrophils that were pre‐incubated with IL‐10 reduced their production of IL‐1β, IL‐6, IL‐8 and TNF‐α (Rigonato‐Oliveira et al., 2018). Together, this suggests that increased IL‐10 concentration by aerobic exercise training could significantly ameliorate lung inflammation and slow progression of CS‐induced lung disease (Figure 1b).

In summary, aerobic exercise training emerges as a promising strategy for modulating the systemic inflammatory response associated with smoke exposure, offering potential therapeutic avenues for alleviating smoke‐induced lung inflammation and its associated complications. Reduction in inflammatory mediators by exercise is expected to be associated with improved lung function and reduced risk of pulmonary infection.

### 6.4 Effect of exercise training on oxidative stress and antioxidant system

Anti‐inflammatory effects of regular exercise training in CS‐exposed mice may also relate to an improved antioxidant defence system (Menegali et al., 2009; Rodrigues Brandao‐Rangel et al., 2017; Surh et al., 2005; Toledo et al., 2012; Toledo‐Arruda et al., 2017). Oxidant–antioxidant and protease–antiprotease imbalance is a central process associated with progression of smoke‐induced lung disease (Cosio & Agustí, 2010; Toledo‐Arruda et al., 2017). Oxidative stress itself plays a critical role in pathogenesis of CS‐induced lung injury and COPD (Rahman & Adcock, 2006). Through activation of redox‐sensitive transcription factors such as activator protein‐1, activating transcription factor 2 (ATF‐2), CREB‐binding protein and NF‐κB, oxidative stress activates several inflammatory signalling pathways, including stress kinase signalling pathways (Rahman & Adcock, 2006; Surh et al., 2005). Consistent with the notion that exercise training ameliorates pulmonary oxidative stress in smoke‐exposed mice, Menegali et al. (2009) showed that superoxide dismutase (SOD) was increased and superoxide concentration was reduced in response to swimming exercise training (30 min twice/day, 5 days/week, 8 weeks) in CS‐exposed mice (Menegali et al., 2009). Similarly, ROS and 8‐isoprostane were found to be lower in BALF and lung tissue of exercise‐trained CS‐exposed mice, compared with CS exposure alone (Toledo et al., 2012; Nemmar et al., 2018). Exercise‐trained mice also appear to be protected against CS‐induced pulmonary oxidative stress. Activity and concentration of SOD and glutathione peroxidase were greater, and catalase (CAT) activity was lower, in CS‐exposed mice with prior exercise training (30 min swimming twice/day, 5 days/week, 8 weeks) compared with CS alone (Nesi et al., 2016). This suggests that the antioxidant defence system is upregulated in lungs of exercise‐trained animals, which may ameliorate effects of oxidant damage induced by CS exposure.

### 6.5 Effect of exercise training on emphysema progression

Murine evidence suggests that aerobic exercise training slows progression of detrimental airway remodelling and lung emphysema in CS exposure (Kubo et al., 2019; Menegali et al., 2009; Rodrigues Brandao‐Rangel et al., 2017). CS exposure leads to alveolar septal destruction, enlargement of alveoli and presence of alveolar macrophages, but these effects were reduced when combined with exercise training (Kubo et al., 2019; Menegali et al., 2009; Rodrigues Brandao‐Rangel et al., 2017; Toledo et al., 2012). Alveolar enlargement and collagen fibre accumulation were found in airway walls in CS‐exposed mice, compared to controls, exercise training alone or combined CS and exercise training groups (treadmill exercise at 50% maximal velocity, 25° incline, 60 min/day, 5 days/week, 8 weeks) (Rodrigues Brandao‐Rangel et al., 2017; Toledo et al., 2012). Most studies deliver CS exposure and exercise training interventions together, suggesting that adding exercise during smoke exposure may protect from detrimental CS effects. Additionally, some studies suggest that exercise‐trained animals are less susceptible to alveolar enlargement caused by CS exposure than those exposed to CS in the untrained state (Kubo et al., 2019; Menegali et al., 2009; Toledo et al., 2012; Toledo‐Arruda et al., 2017). This suggests that fitness itself, rather than the act of exercising per se, may provide protection against CS‐induced inflammation and alveolar destruction. Taken together, experimental studies in mice suggest that aerobic exercise training slows progression of emphysema caused by CS exposure.

## 7 EVIDENCE FROM HUMAN STUDIES

PR, in which exercise training is the major component, is one of the most effective therapies for improving COPD symptoms and outcomes (Casaburi & ZuWallack, 2009; Holland et al., 2021; Rochester et al., 2023; Spruit et al., 2013). Direct effects of exercise training include increased muscle mass and strength, muscle oxidative capacity and capillarity, and cardiovascular function, which together reduce reliance on substrate level phosphorylation and ventilatory demands of exercise (Casaburi & ZuWallack, 2009). This allows a slower and deeper breathing pattern, less dynamic lung hyperinflation, ameliorates dyspnoea on exertion and leads to increased exercise tolerance (Spruit et al., 2013). Less obvious, but arguably equally beneficial, effects of PR in COPD include reduced anxiety and depression, improved glucose and lipid control, reduced adiposity and amelioration of systemic inflammation. For example, increased muscle strength and cross‐sectional area following PR in COPD might be partly attributable to reduced inflammation, as systemic inflammation inhibits anabolism and stimulates catabolism (van der Vlist & Janssen, 2010). Despite this, the mechanisms mediating any association between these physiological effects of PR and the observations that PR reduces hospital readmissions, AECOPD and mortality are unknown.

While there is a reasonable biochemical and physiological basis to support the hypothesis that exercise can modulate the chronic inflammatory response in COPD, this has proven difficult to demonstrate in COPD patients. It remains challenging to determine the impact of PR on inflammatory status in COPD for many reasons including wide variations in PR programme components, inflammatory assessment methodologies, and characteristics of patients entering PR programmes.

Exercise training is an essential component of a comprehensive PR programme (Holland et al., 2021). PR programmes also have a large degree of discretion when tailoring exercise prescription to individual patients. Thus, there are numerous factors in exercise programmes that could confound studies examining chronic inflammatory response to PR. Sources of variation include exercise duration (7–20 weeks), whether the programme is centre‐based (either inpatient or outpatient) or delivered remotely in the home (including virtually), exercise frequency (once to thrice weekly), exercise type (cycling, walking, upper vs. lower extremity training, endurance or strength training, balance training, physical activity promotion), and exercise intensity. In several studies evaluating inflammatory responses, exercise intensity was not specified, while other studies prescribed training based on detailed knowledge of baseline cardiopulmonary exercise tests and strength or power tests. These variations raise the possibility that some exercise prescriptions were insufficient to achieve sustained modulation of the inflammatory system. On the other hand, it is known that intensive exercise bouts could induce a systemic immunological response and oxidative stress, which might increase inflammation in patients (Madani et al., 2018), although promotion of pro‐inflammatory effects by exercise has only been observed in muscle wasted COPD patients and then only after acute bouts of intense exercise (Van Helvoort et al., 2006).

As previously noted, this review focuses on tobacco‐related COPD and responses to exercise training. Hence, studies of responses to acute exercise, or those hospitalized or with non‐stable COPD will not be addressed here. Clinical guidelines currently recommend starting PR within 3 weeks of hospital admission for AECOPD (Rochester et al., 2023), and therefore this group tends to be well studied. However, because PR is the standard of care for post‐AECOPD treatment, this complicates design of randomized controlled trials where randomization to no‐treatment lacks equipoise. Therefore, many studies focusing on exercise training and the immune system enrol stable COPD patients in the absence of recent AECOPD. It is unclear whether results from this population can be appropriately extrapolated to post‐hospitalization individuals. Wait‐list trials or trials involving ‘usual care’, where usual care includes the large majority (>95%) of participants who opt not to participate in PR within 90 days of discharge (Lindenauer et al., 2020), may provide the opportunity to construct appropriate study designs. There is less evidence available for approaches such as early rehabilitation (PR that starts during hospitalization for AECOPD), and hence these will not be discussed here. We also note that the study of exercise‐induced immunomodulation in COPD patients, as other conditions, is typically limited to measurement of circulating, and rarely BAL fluid, variables.

Multiple studies examined the impact of PR and/or exercise training on the inflammatory state in stable COPD patients; however, results are inconsistent, possibly because of methodological variability described above. While in general, chronic exercise training seems to reduce systemic and BALF inflammatory mediators in COPD patients (do Nascimento et al., 2015; Sciriha et al., 2017; Silva et al., 2018a, 2018b; Szczegielniak et al., 2011; Uzeloto et al., 2022; Wang et al., 2014), some studies show no change (Bolton et al., 2007; Neunhäuserer et al., 2021; Petersen et al., 2008; Rabinov et al., 2003; Rodriguez et al., 2012; Ryrsø et al., 2018; Sciriha et al., 2017; Thyregod et al., 2022; Valero‐Breton et al., 2023; Vogiatzis et al., 2007) (Table 1). Some variability may be related to timing of post‐training blood sampling; in some cases, blood was sampled weeks after completing a PR course, while in others, samples were collected immediately following a post‐training exercise test, with latency of sample collection varying from immediate (at peak exercise) to up to several hours later. Post‐training exercise tests also ranged from submaximal to incremental cycle ergometer tests to exhaustion tests. Overall, it is unclear whether an exercise training programme that elicits a robust training response in COPD (e.g., assessed by increased exercise tolerance) is accompanied by sustained amelioration of blood or BALF cytokine or chemokine concentration.

Patient selection and sample size also contribute to wide variability in available data. COPD is a broad category of airway and parenchymal lung diseases that is defined by presence of expiratory flow limitation. Pulmonary features, together with presence or absence of low skeletal muscle mass or weakness, define different phenotypes and outcomes for COPD patients (Maltais et al., 2014; Mason et al., 2021, 2022). An inflammatory phenotype has also been hypothesized; the ECLIPSE trial reported that 16% of COPD patients maintained persistently elevated serum inflammation over the course of 3 years (Agustí et al., 2012). It is unknown whether COPD patients with more persistent inflammation are a subgroup that would benefit more from hypothesized immunomodulatory effects of exercise training. While there have been efforts to determine whether the inflammatory response in cachectic COPD patients is altered by PR (Van Helvoort et al., 2006; Vogiatzis et al., 2010), no study, to our knowledge, has been adequately designed to investigate effects of exercise on COPD patients with the proposed inflammatory sub‐phenotypes. It is also often unclear whether studies have excluded or controlled for individuals who are taking inhaled or systemic corticosteroids or have other medications or comorbidities that could alter immune response.

### 7.1 Exercise training and immune cell number

Unfortunately, few studies have evaluated response of white blood cell number to exercise training in COPD patients (Neunhäuserer et al., 2021; Ryrsø et al., 2018; Sciriha et al., 2017) (Table 1). At baseline, COPD patients commonly have high leukocyte numbers, mainly due to neutrophilia, compared to healthy control individuals (Ryrsø et al., 2018). However, in contrast to preclinical studies, neither short‐term (Neunhäuserer et al., 2021; Ryrsø et al., 2018) nor longer‐term (Sciriha et al., 2017) exercise programmes of different types and intensities that are prescribed for people with COPD have successfully modified white blood cell counts.

### 7.2 Effect of exercise training on inflammatory mediators

Typically, baseline circulating inflammatory mediators are greater in COPD patients than healthy controls (Petersen et al., 2008; Rabinov et al., 2003; Ryrsø et al., 2018). Despite this, responses to exercise training of inflammatory mediators such as CRP, TNF‐α, IL‐6 and IL‐8 are not consistent among reports (Table 1), and these differences do not seem to be related in a simple fashion to baseline degree of systemic inflammation (Spruit, Gosselink et al., 2005). Despite these significant limitations, some trends are evident in the literature.

CRP is the most extensively studied biomarker in COPD in response to exercise training. CRP is an acute‐phase protein induced by IL‐6 and TNF‐α during systemic inflammation and has been associated with poor physical performance and muscle strength in elderly people (Cesari et al., 2004). Elevated CRP and fibrinogen are associated with increased mortality in COPD patients (Celli et al., 2019; de Torres et al., 2008). Cross‐sectional studies show that CRP concentration is related to clinical outcomes, such as exercise tolerance, health status and AECOPD (Ferrari et al., 2013). In general, high rates of physical activity and long‐term (9 months) exercise training are associated with lower concentration of CRP in healthy populations (Abramson & Vaccarino, 2002; Geffken et al., 2001; Kasapis & Thompson, 2005; Lakka et al., 2005; Mattusch et al., 2000). However, findings on CRP response to exercise training in COPD are inconsistent. Some studies find no change in CRP concentration following exercise training (Jenkins et al., 2020; Neunhäuserer et al., 2021; Petersen et al., 2008; Ryrsø et al., 2018; Thyregod et al., 2022; Van Helvoort et al., 2006; Vogiatzis et al., 2010), whereas others report significant reduction (Kantorowski et al., 2018; Márquez‐Martín et al., 2014; Sciriha et al., 2017; Wang et al., 2014). It appears that studies reporting substantial decrease in CRP levels in COPD patients tend to encompass longer training duration (on average, 15 weeks vs. 8 weeks). In line with this, Wang et al. (2014), using 6 months of mobile‐based home exercise training, showed that circulatory CRP concentration was decreased in COPD patients after 2 months and remained low for the remainder of the study (Wang et al., 2014). Baseline CRP may also be important in interpreting training response. Recent findings of the large HERITAGE Family Study of exercise training in 652 healthy people suggests that 20 weeks of endurance exercise training reduced CRP the most in those with greatest baseline CRP (Lakka et al., 2005). Given its association with mortality in COPD, CRP may be a promising treatable biomarker for anti‐inflammatory efficacy of PR. Fibrinogen is another acute‐phase inflammatory protein that is established as a surrogate biomarker for COPD exacerbation (Duvoix et al., 2013). It is noteworthy that only two studies have examined the impact of exercise training or PR on fibrinogen, but that both found a significant decrease (Jenkins et al., 2020; Neunhäuserer et al., 2021). This is consistent with benefits of PR on reducing AECOPD and mortality (Puhan et al., 2016).

TNF‐α is another extensively studied cytokine in COPD in response to PR (Neunhäuserer et al., 2021; Petersen et al., 2008; Rabinov et al., 2003; Silva et al., 2018a, 2018b; Thyregod et al., 2022; Valero‐Breton et al., 2023; Vogiatzis et al., 2007; Wang et al., 2014). Although most studies show that resting serum TNF‐α does not change following PR or exercise training (Bolton et al., 2007; Rabinov et al., 2003; Vogiatzis et al., 2010, 2007), one study focusing on strength training found significant decrease in serum TNF‐α after 12 weeks (Silva et al., 2018). This study also reported significant decrease in IL‐6 and IL‐15 after resistance exercise training (Silva et al., 2018). Endurance exercise training in COPD, on the other hand, had no effect on plasma TNF‐α or TNF‐α gene expression (Rabinov et al., 2003). In general, lung tissue concentration of TNF‐α in COPD patients is greater than in health (Table 1, Figure 1a), which raises the question of whether lack of systemic TNF‐α response to exercise training in COPD is primarily attributable to ‘spill over’ from the lungs and whether this spill over is largely unaffected by exercise training. In line with this, Gielen et al. (2003) found no change in serum levels of TNF‐α and IL‐6 in chronic heart failure (CHF) patients following longer‐term exercise training (70% ${\dot{V}}_{O_{2}\max}$, 20 min/day, 6 months), while skeletal muscle TNF‐α and IL‐6 were significantly decreased (Gielen et al., 2003). In addition, studies investigating effect of exercise on TNF‐α in healthy and athletic individuals have also shown no change in circulatory concentration of TNF‐α following acute exercise or exercise training (Walsh et al., 2011). It appears that further work is needed, perhaps using endotoxin‐stimulated blood or cells (Abbasi et al., 2013) in several tissue types (blood, BALF, muscle), to determine whether exercise training in COPD has a beneficial effect on TNF‐α and its disease‐related targets.

The response of IL‐6 to exercise training in COPD is also controversial and not impressive. IL‐6 concentration was either reduced (da Silva et al., 2017; Kantorowski et al., 2018; Silva et al., 2018) or unchanged (Bolton et al., 2007; do Nascimento et al., 2015; Petersen et al., 2008; Rabinov et al., 2003; Ryrsø et al., 2018; Uzeloto et al., 2022; Van Helvoort et al., 2006; Vogiatzis et al., 2007; Wang et al., 2014) after training in COPD (Table 1). Of note in one of these studies, COPD patients not taking part in the exercise programme increased IL‐6 at 3 and 6 months compared to the exercise training group that remained unchanged (Wang et al., 2014). Considering that IL‐6 and TNF‐α can stimulate each other via feedback loop mechanisms, and TNF‐α seems not to respond to training in COPD, the lack of a clear training effect on IL‐6 is perhaps understandable. Nevertheless, there is poor consistency in the reporting, prescription, adherence and physiological characteristics of participants in these studies, which complicates understanding of variation in IL‐6 response to PR in COPD. While chronically elevated IL‐6 and CRP reflect ongoing local TNF‐α production and inflammation (Petersen et al., 2008), IL‐6 also has an anti‐inflammatory effect when released from muscle during exercise through a TNF‐α‐independent pathway (Steensberg et al., 2003). As a result, IL‐6 stimulates production of anti‐inflammatory cytokines such as IL‐10, which inhibit production of the inflammatory cytokine TNF‐α (Steensberg et al., 2003). Therefore, general failure to see robust reductions in IL‐6 following PR could be attributable to reduced ability of COPD patients to engage a large muscle mass in exercise (due to ventilatory limitation), insufficient exercise intensity and/or training volume (Petersen et al., 2008).

The literature is more consistent in showing a decrease in circulating IL‐8 (CXCL8) in COPD patients following exercise training (da Silva et al., 2017; do Nascimento et al., 2015; Gelinas et al., 2017; Márquez‐Martín et al., 2014; Spruit et al., 2005; Szczegielniak et al., 2011; Uzeloto et al., 2022; Wang et al., 2014) (Table 1, Figure 1b). IL‐8 is a macrophage‐released chemokine observed in COPD during early stages of lung inflammation, and plays a crucial role in attracting neutrophils and monocytes to lung tissue (Donnelly & Barnes, 2006). Concentration of IL‐8 in sputum and BALF of COPD patients is significantly greater than in health and correlates with increased mucus production and airway remodelling (Donnelly & Barnes, 2006; Vitenberga et al., 2019). Therefore, exercise‐training‐related reduction in IL‐8 would be expected to contribute to reduced chronic pulmonary inflammation and perhaps improve clinical status of COPD patients. During a comprehensive home‐based PR study, participants in the exercise training group (80% ${\dot{V}}_{O_{2}\mathrm{peak}}$, 2 h exercise, 2 days/week) demonstrated substantial decrease in plasma IL‐8 concentration at 2, 3 and 6 months, in comparison to baseline, whereas IL‐8 in the untrained control group increased progressively over time (Wang et al., 2014). Plasma IL‐8 was also reduced following 8 weeks of home‐based PR (85% ${\dot{V}}_{O_{2}\mathrm{peak}}$, 3 days/week) (do Nascimento et al., 2015) or 12 weeks of centre‐based combined resistance and endurance exercise training (Márquez‐Martín et al., 2014). In addition, IL‐8 expression in CD4^+^ T lymphocytes was significantly reduced after combined resistance and endurance training compared to before training in stable COPD patients (Uzeloto et al., 2022). These results suggest that IL‐8, as a disease‐worsening factor, may be an essential mediator in assessing inflammatory status of stable COPD patients in response to PR or exercise training.

Limited data are available about response of some other inflammatory mediators to PR or exercise training in COPD patients (Table 1). IL‐18, ICAM‐1 and VCAM‐1 did not change after exercise training (Petersen et al., 2008; Gelinas et al., 2017; Ryrsø et al., 2018), but in one study of 35 participants, IL‐10 was increased and IL‐15 had a tendency to increase in response to acute exercise after exercise training (Silva et al., 2018). IL‐15 is a pluripotent anti‐apoptotic cytokine implicated in the antiviral immune response, and regulates cell proliferation, survival and function of NK cells (Budagian et al., 2006). Plasma IL‐15 is lower in smokers and in emphysema (de‐Torres et al., 2013; Mian et al., 2009), and there is a moderate positive correlation between IL‐15 and forced expiratory volume in 1 s (FEV_1_)/forced vital capacity (FVC) in mild–moderate COPD, but not in severe COPD (Silva et al., 2018). No other study has confirmed the increase of systemic IL‐15 by exercise training in COPD patients. IL‐10, on the other hand, selectively blocks expression of pro‐inflammatory genes encoding TNF‐α and IL‐8 in myeloid cells (Moore et al., 2001), suggesting an important role in resolution of inflammation. Serum IL‐10 is lower in stable COPD and is positively associated with lung function (Huang et al., 2016; Mian et al., 2009; Silva et al., 2018). In addition, lower IL‐10 is associated with COPD severity and a greater AECOPD frequency (Silva et al., 2018a, 2018b; Sun et al., 2013). Therefore, considering that IL‐10 selectively suppresses LPS‐induced expression of IL‐8 and TNF‐α (Castellucci et al., 2015), lower IL‐10 or its activity may contribute to slowing of COPD progression. While evident from animal studies that exercise increases IL‐10 concentration, further well‐defined human studies are required to better understand how IL‐10 responds to exercise training in COPD patients.

### 7.3 Effect of exercise training on oxidative stress

Oxidative stress is considered as a contributory mechanism in development of COPD (Mercken et al., 2005). Inflammatory cytokines in COPD are associated with reduced anti‐oxidant activity and increased ROS production by mitochondria and neutrophils in COPD (Reid & Li, 2001). ROS also activate redox‐sensitive transcription factors that control gene expression of inflammatory mediators such as IL‐6 (Reid & Li, 2001). The exercise‐trained state tends to reduce ROS production (Alcazar et al., 2019; Watson et al., 2021); however, not many studies have evaluated effects of exercise training on systemic oxidative stress and ROS production in COPD patients. Mercken et al. (2005) demonstrated that 8 weeks of intensive supervised PR that yielded an increased exercise capacity was associated with decreased ROS‐induced DNA damage after a submaximal exercise test in stable COPD patients (Mercken et al., 2011). A few other studies have demonstrated decreased plasma protein carbonyls and xanthine oxidase (XO) after 8–12 weeks of aerobic exercise training in COPD patients (Pinho et al., 2007; Alcazar et al., 2019). In addition, Nesi and colleagues (2016) found that following exercise training in water (2 × 30 min/day, 5 days/week, 8 weeks), the concentration of SOD and glutathione peroxidase (GPx) activity in lung tissue were higher in exercise+CS mice than CS mice (Nesi, de Souza et al., 2016). These studies suggest that exercise training is associated with decreased oxidative stress in COPD patients. The mechanisms by which exercise influences oxidative stress in COPD patients may include increasing antioxidant activity, decreasing oxidase activity and improving mitochondrial function (Ryrsø et al., 2018; Van Helvoort et al., 2006; Zhang et al., 2021).

## 8 CONCLUSION

Experimental preclinical studies show clear anti‐inflammatory response of exercise training in smoke‐exposed mice. In COPD patients, however, available data regarding effect of PR or exercise training on inflammatory status are inconsistent. Although the effect of exercise training on circulating CRP and IL‐8 favours reduction, IL‐6 and TNF‐α were generally unaltered by exercise training. Possible explanations for variability in immunomodulation by exercise training in COPD include: severity and phenotype of COPD (predominant airways inflammation vs. emphysema) (Spruit et al., 2005; Troosters et al., 2001); variation in baseline inflammatory state; baseline fitness and/or variability in response of fitness to training; and types (strength vs. endurance), intensity, frequency and duration of exercise training. Whereas endurance exercise training benefits all severities of COPD in terms of increased exercise endurance and reductions in dyspnoea (Casaburi et al., 2024), it seems that some patients may gain greater immunomodulatory benefits than others. Whether this benefit is associated with observed reduction in exacerbation rates following PR awaits further research.

## AUTHOR CONTRIBUTIONS

Asghar Abbasi and Harry B. Rossiter conceived this project. Asghar Abbasi and David Wang drafted the manuscript, which was finalized by Richard Casaburi, William W. Stringer, and Harry B. Rossiter. All authors have read and approved the final version of this manuscript and agree to be accountable for all aspects of the work in ensuring that questions related to the accuracy or integrity of any part of the work are appropriately investigated and resolved. All persons designated as authors qualify for authorship, and all those who qualify for authorship are listed.

## CONFLICT OF INTEREST

H.R. reports consulting fees from the NIH RECOVER‐ENERGIZE working group (1OT2HL156812), and is involved in contracted clinical research with Astellas, GlaxoSmithKline, Genentech, Intervene Immune, Mezzion, Novartis, Regeneron, Respira and United Therapeutics. He is a visiting Professor at the University of Leeds, UK. The other authors declare no conflicts of interest.
